# Adsorption Effect and Adsorption Mechanism of High Content Zeolite Ceramsite on Asphalt VOCs

**DOI:** 10.3390/ma15176100

**Published:** 2022-09-02

**Authors:** Wei Chen, Hui Zhao, Yongjie Xue, Xiwen Chang

**Affiliations:** 1 School of Art, Hubei University of Education, Wuhan 430001, China; 2 State Key Laboratory of Silicate Materials for Architectures, Wuhan University of Technology, Wuhan 430070, China

**Keywords:** zeolite ceramsite, volatile organic compounds, asphalt materials, adsorption mechanism, BET analysis

## Abstract

In order to meet the requirements of industrial-scale fixed beds and develop an excellent adsorbent for asphalt VOCs. Zeolite ceramsite containing binder was prepared and successfully applied to the inhibition of asphalt VOCs. The results showed that prepared zeolite ceramsite possessed a high degree of crystallinity, and its main crystal phase is zeolite. The micropores with a pore size of 0.88 nm dominated the pore size distribution of the material. The adsorption experiment of asphalt VOCs showed a lower VOCs adsorption effect of 8.72% at a small dosage of 5%, while at a large dosage of 50%, the adsorption effect of VOCs exceeded 45%. This might be caused by the quite small external specific surface area, which occupied only 8.3% of the total specific surface area, and the low intraparticle diffusion coefficient due to the micropores. Meanwhile, the kinetics diameters of most aromatic hydrocarbons, which were comparable to the pore size of micropores, and the increase in the intraparticle diffusion resistance of aliphatic hydrocarbon molecules were the important factors in obtaining high adsorption of aromatic hydrocarbons in asphalt VOCs. Furthermore, the results indicated that the particulate adsorbent with a microporous structure should be mixed into the asphalt as a fine aggregate rather than an asphalt modifier for better asphalt VOCs adsorption effect.

## 1. Introduction

Asphalt is a viscoelastic material and is most widely used for highway road construction in China. With road construction developing rapidly in recent years, the demand and consumption of asphalt are increasing dramatically. A significant quantity of VOCs is emitted into the environment when manufacturing, transporting, and building asphalt pavement. Asphalt VOCs contain various harmful components, especially polycyclic aromatic hydrocarbons (PAHs), which are recognized as the most carcinogenic compounds [1,2]. Meanwhile, prolonged exposure to asphalt VOCs can cause severe respiratory and nervous system diseases [3,4]. It can be said that asphalt VOCs are more toxic and carcinogenic than others derived from industrial production activities, which have seriously affected human health and the natural environment. Therefore, reducing VOCs released from asphalt has become a popular topic [5,6].

In recent years, two kinds of effective technologies have been proposed to lessen the emission of asphalt VOCs. One is warm mixing technology by lowering the construction temperature [7], and the other is adding emission reduction agents to hot mix asphalt mixture (HAM). HAM is still widely used in most asphalt pavements for its great engineering performance. Thus, the later removal technology gets more attention for effectively reducing the asphalt VOCs. Asphalt VOCs’ emission reduction agents can be divided into two categories according to the releasing mechanism. The first one is polymer modifiers. The introduction of polymer modifiers makes the internal structure of the asphalt binder become more compact. The asphalt molecules, especially the light components, are fixed in the network structure through the formation of reticular structure, thereby reducing the emission of asphalt VOCs [8]. The second one is adsorbents, which are regarded as one of the mainstream techniques due to their low energy consumption, facile operation of adsorption processes, and low operation cost [9,10]. Many researchers have explored the nanoparticle [11,12], activated carbon [13,14], and metal organic framework [15] as adsorbents for the adsorption application of benzene and its homologs (representative components in asphalt VOCs) and evaluated the kinetic model for the whole adsorption process. However, due to the complexity of asphalt VOCs, it is quite difficult to evaluate the kinetic model to predict the adsorption behavior using conventional methods. Previous research on asphalt VOCs adsorbents mainly included three aspects: the development of adsorbents, the evaluation of the adsorption effects, and the revelation of adsorption mechanisms. Long et al. [16] reported a significant asphalt VOCs inhibitory effect with activated carbon as adsorbent filler and revealed that adding activated carbon to asphalt binder would maintain good storage stability. Zhou et al. [17] investigated biochar’s adsorption effects and mechanism on the VOCs from asphalt. The asphalt VOCs emissions were reduced owing to the porous structure of biochar which adsorbed a variety of saturated hydrocarbons. Tang et al. [6] employed geopolymer additives to reduce VOCs emissions of warm-mix asphalt. The results showed that a geopolymer with slit-shaped mesopores could serve as a promising adsorbent. Motivated by those studies mentioned before, developing new adsorbents for reducing the emission of asphalt VOCs is of great significance. The previous study demonstrated that spent fluid catalytic cracking (FCC) catalysts as sorbents led to a suppression effect of asphalt VOCs, especially for the PAHs [18]. It is noted that most researchers focus on the adsorption effects of powdered additives. Studies on granular additives’ effects on the adsorption performance of asphalt VOCs have rarely been involved.

Due to its extensiveness of pore regulation, uniformity of pore distribution, and thermal stability at high temperatures, zeolite is a class of highly porous materials used widely in the realms of catalysis, adsorption, and separation [17,19,20,21]. It is worth mentioning that zeolite powder has been explored as an excellent modifying filler to remove VOCs generated from asphalt [22,23]. Nevertheless, commercial zeolite molecular sieves generally require the agglomeration of the material into particles of suitable size and shape owing to hydrodynamic constraints in the reactors of industrial-scale fixed beds [24]. These particles are produced by mixing powdered zeolite with a binding or cementing clay, and then the zeolite ceramsite with high mechanical strength is formed by further drying and calcination. Ceramsite, a typical porous material, may be classified into many varieties based on the raw materials used. Moreover, because of the lightweight and low thermal conductivity properties, ceramsite can be incorporated into asphalt mixture as fine aggregates to reduce the self-weight and improve the thermal resistance ability of asphalt mixture [25]. Therefore, the zeolite ceramsite might be a promising lightweight fine aggregate for removing VOCs in asphalt binders. However, most studies have focused on preparing zeolite ceramsite from industrial waste and the application of zeolite ceramsite in concrete [26,27,28]. To our understanding, quite a limited investigation has been done on the adsorption effect and mechanism of zeolite ceramsite on asphalt VOCs. Consequently, it is of great theoretical and practical significance to implement the adsorption exploration of zeolite ceramsite in the aspect of asphalt VOCs. Commercial 13X zeolite powder was selected as basic raw material considering the complexity and diversity of asphalt VOCs.

In this present work, the 13X zeolite ceramsite containing attapulgite clay binder was used as the research object to investigate its potential reduction of VOCs in asphalt binders through Thermal Desorption-Gas Chromatography-Mass Spectrometry (TD-GC-MS). Meanwhile, to explain the mechanism of VOCs suppression and adsorption, scanning electron microscope (SEM), X-ray diffractometer (XRD), X-ray fluorescence spectrometer (XRF), Fourier transform infrared spectrometer (FTIR) and specific surface area and porosity analyzer were employed to characterize the basic properties of zeolite ceramsite.

## 2. Materials and Methods

### 2.1. Materials

#### 2.1.1. 13X Zeolite and Attapulgite Clay

Commercial 13X zeolite was purchased from a water purification material sales company in Henan Province. The attapulgite clay tested in this study was obtained from Xuyi Mine, Jiangsu Province, PR China.

#### 2.1.2. Asphalt Binder

The Pen 60/80 matrix asphalt from Wuhan Municipal Construction was used to study the suppression of asphalt VOCs resulting from the zeolite ceramsite, and its basic performances were concluded in Table 1.

### 2.2. Methods

#### 2.2.1. Preparation of Zeolite Ceramsite

The granulation process of zeolite ceramsite was carried out in a rotary tilted disc of 400 mm diameter and 100 mm depth, using attapulgite clay as a binder. Before granulation, zeolite and binder were ground separately in a ball mill and then sieved to collect 200 mesh passing powder. Subsequently, dried at 105 °C for 12 h and stored in a sealed container for further processing. During the granulation process, 500 g of a mixture of 80% zeolite and 20% attapulgite clay was homogenized in a hermetic vessel for 10 min. The mixture was loaded into the granulator operating at the inclination of 45° and rotating velocity of 55 rpm. Meanwhile, agitation was started, and the required amount of deionized water was added at about 10 mL/min by spraying for up to 25 wt.% water content in the wet paste. When the water addition was finished, the granules were unloaded from the disc and sieved to collect particles between meshes 8 and 12 (2.36 and 1.70 mm, respectively). Finally, the obtained particles were air-dried for about 24 h and then calcined in an electrical tube furnace with an air atmosphere at 600–700 °C for 2 h to obtain zeolite ceramsite (ZC).

#### 2.2.2. Preparation of Asphalt Mortar

The tank with 200 g asphalt binder was heated at 135 °C, and zeolite ceramsite was blended. The incorporation ratios were designed here in two different ways. The first was the way of asphalt modification, zeolite ceramsite was added as a modifier of asphalt, and the incorporation ratio was 5%, which was marked as 5ZC. The second was the way of asphalt aggregate, zeolite ceramsite performed as a fine aggregate of asphalt binder, and the incorporation ratio is 50%, which was marked as 50ZC. It is worth mentioning that the dosage of zeolite ceramsite respectively stemmed from Wu et al. [22] and Che et al. [25], owing to their optimal costs and performance. To mix evenly, the mixtures combined with zeolite ceramsite of different incorporation ratios were kept stirring and shearing at a speed of 500 rpm in the mixer for 30 min at a temperature of 135 °C with a shearing instrument. The matrix asphalt, which can be equivalent to 0% added filler, marked as 0ZC, also experienced the same preparation process. Then the mixtures were cool down to room temperature for the subsequent experiments.

#### 2.2.3. Characterization of Zeolite Ceramsite

The main properties, such as specific surface area, pore volume, pore size distribution, and structural characterization of the prepared zeolite ceramsite, are crucial to exploring the VOCs removal mechanism. The ASAP-2020 automatic specific surface area and porosity analyzer were used to analyze the pore structure of the prepared zeolite ceramsite with the BET analysis. The XRD (Empyrean, Malvern Panalytical, Almelo, The Netherlands) was used to detect the main phases in raw materials and prepared products and to explore the changes in the crystal types during the preparation. X-rays with a wavelength of 1.54 Å were produced by Cu Kα radiation (40 kV, 40 mA) and filtered using a Nickel filter. The chemical compositions of raw materials and prepared zeolite ceramsite were verified by semiquantitative X-rays fluorescence (XRF) analysis using a Zetium XRF spectrometer (Malvern Panalytical, The Netherlands) with a 4 kW maximum capacity. The FTIR (Nicolet 6700, Thermo Fisher Scientific, Waltham, MA, USA) was used for functional group detection of zeolite ceramsite. The FTIR resolution was 4 cm^−1,^ and the spectrum scan frequency was 20 times per minute, with a spectrum region ranging from 4000 cm^−1^ to 400 cm^−1^. As for microscopic morphology, the field emission scanning electron microscope with Quanta FEG 450 (FEI, Hillsboro, OR, USA) was used to observe the surface morphology of the prepared product.

#### 2.2.4. Adsorption Experiment of Asphalt VOCs

**Figure 1:**
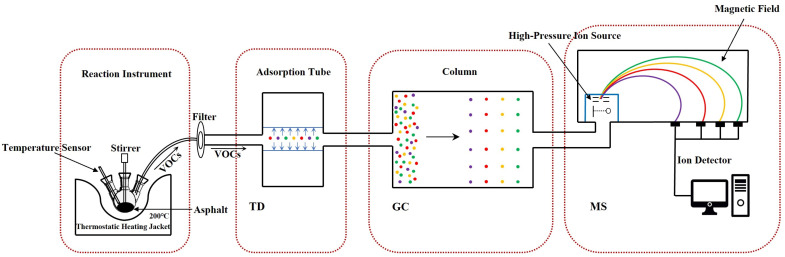
The operation procedure of TD-GC-MS in VOCs.

As shown in Figure 1, the compositions and content of VOCs were obtained through the TD-GC-MS test. Generally, three components of VOCs generation, VOCs collection by adsorption tube and desorption detection analysis, compose the whole operation process. In the reaction instrument of VOCs generation, the flask containing the sample mass of 100 g was heated to 160 °C and kept the temperature for 30 s. During this period, the exhaust port was closed to prevent VOCs from overflowing. After that, adsorption was started with an adsorption tube with a capacity of 100 mL, and the adsorption was carried out at a flow rate of 100 mL/min, controlled by an airflow meter for 30 s. Once the adsorption was over, the adsorption tube was taken out and sealed immediately. Three samples of 0ZC, 5ZC, and 50ZC repeated the above operation under the same test conditions. The VOCs stored in the adsorption tube can be desorbed by the TD system and then sent to the GC analyzer, which can perform the component separation. The MS instrument will then be used to evaluate the composition of VOCs, which can accurately perform VOCs composition analysis [4].

## 3. Results and Discussion

### 3.1. Basic Characterization of Adsorbent

#### 3.1.1. Chemical Composition Analysis

The chemical compositions of the obtained zeolite, attapulgite clay and prepared zeolite ceramsite are listed in Table 2 through XRF analysis. An attapulgite clay possesses the typical characteristic of high magnesium and iron content, which may also be used to track the final content of the binder contained inside the zeolite ceramsite. The zeolite raw material, except for the high content of silicon, aluminum, and sodium, contains almost no other impurities. On the contrary, attapulgite clay contains more potassium or other ions (such as magnesium, calcium, and iron). It is important to note that during the preparation of zeolite ceramsite, ions other than sodium might degenerate the zeolite crystals, changing the pore size and then impairing the capability of adsorption [29]. The zeolite ceramsite prepared from attapulgite clay and the commercial 13X zeolite show similar chemical compositions. Still, it contains traces of iron, magnesium, and calcium oxides that descend from the attapulgite clay.

#### 3.1.2. XRD Results

**Figure 2:**
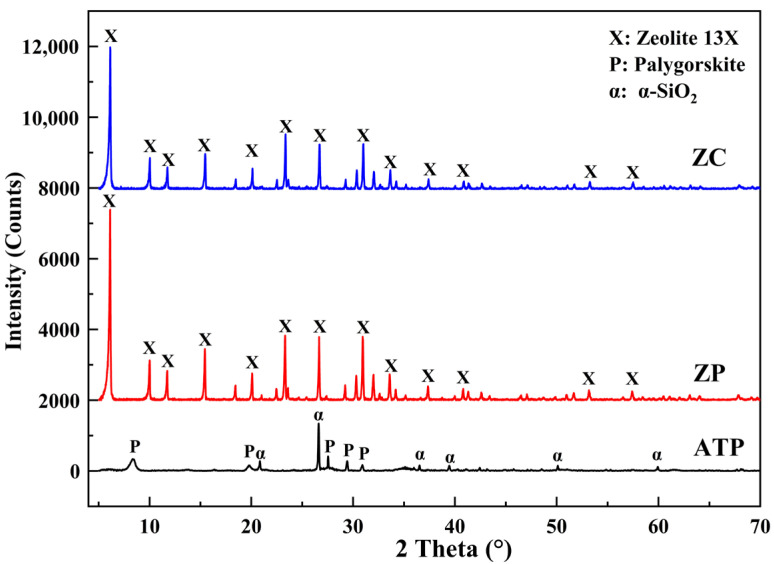
XRD spectra of raw materials (ZP and ATP) and prepared ceramsite (ZC).

Figure 2 shows the XRD spectra of three samples, i.e., commercial 13X zeolite powder marked as ZP, attapulgite marked as ATP, and zeolite ceramsite marked as ZC. With a high degree of crystallinity, the main mineral components of ATP clay are palygorskite and α-quartz, in which quartz impurities are shown by its most intense peak situated at 2θ = 26.6° (d_101_ = 0.335 nm) [30]. The diffraction peaks of ZP are typical of FAU-type NaX zeolite (JPCDS card no.38-0237) without additional phases [31].

Although 20% ATP clay is incorporated in the preparation process, the final prepared ZC product is not detected the diffraction peaks of palygorskite by the analysis of the XRD. This is because the crystal structure of the palygorskite was destroyed during the calcination process, and the high activity SiO_2_ with unstable crystallinity was formed [32]. The XRD pattern of ZC is almost identical to that of ZP, in which no other crystal phase can be observed for ZC compared with ZP; only the intensities of diffraction peaks are slightly lower than those of ZP. Zhao et al. [33] and Chen et al. [34] also observed this phenomenon and reported the 13X zeolite diffraction peaks consistent with the prepared ZC. Therefore, the main crystal phase in the prepared ZC product with high crystallinity is 13X zeolite, suggesting ZC may have an impressive adsorption potential on asphalt VOCs.

#### 3.1.3. FTIR Analysis

**Figure 3:**
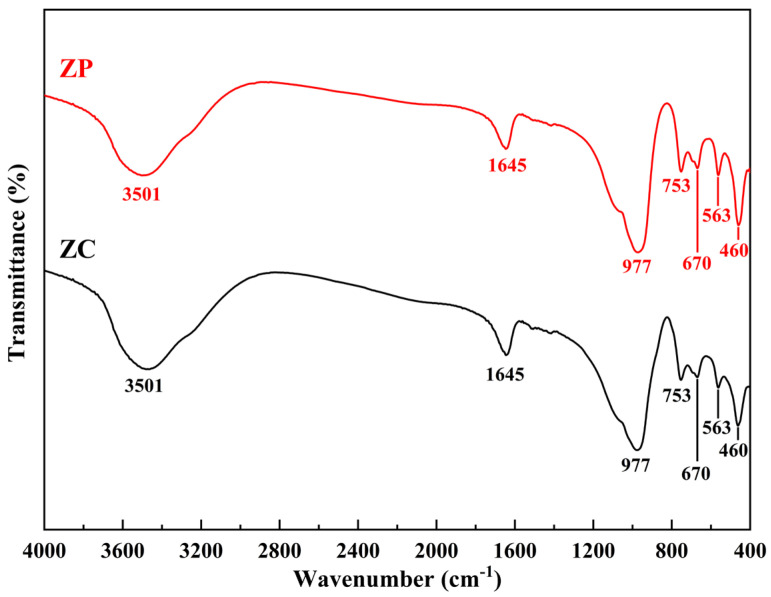
FTIR spectra of samples ZC and ZP.

The FTIR spectra of ZP and ZC are shown in Figure 3. The peaks observed at about 3501 cm^−1^ and 1645 cm^−1,^ respectively, correspond to the OH-stretching and OH-bending vibration of bound water present in the zeolite due to incomplete dehydration of the samples. The absorption peaks at 977 cm^−1^ and 670 cm^−1^ correspond to TO_4_ (where T = Si or Al) tetrahedral asymmetric and symmetric stretch vibration [35]. The appearance of the peak at 563 cm^−1^ is assigned to double six rings (D6R) which are the main building blocks of the octahedral structures in zeolite X [36]. In addition, the absorption peak at 460 cm^−1^ is attributed to T-O bending vibration. The peak at 753 cm^−1^ is connected with the symmetric stretching vibration of Si (Al)-O in the Si-O-Al framework [31,37]. All the characteristic bands of ZC are similar to ZP. FTIR results revealed that the main structures in ZC and ZP are both Si-O tetrahedrons and Al-O tetrahedrons. No obvious absorption peaks are observed for impurity atoms, which is consistent with the XRD analysis.

#### 3.1.4. Microscopic Study

**Figure 4:**
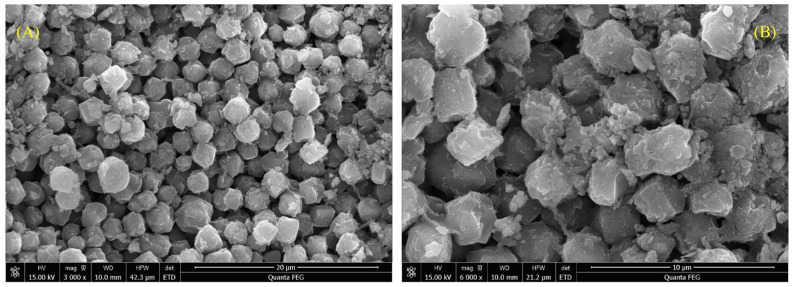
SEM image of ZC at different magnifications: (**A**) 3000×, (**B**) 6000×.

The microscopic morphology of ZC is shown in Figure 4. From the SEM image, it could be found that the size of zeolite particles in prepared ZC is about 2 μm, and these particles are stacked with each other to form various channels.

### 3.2. Adsorption Effect of ZC on VOCs Emission

#### 3.2.1. Analysis of Asphalt VOCs Composition

**Figure 5:**
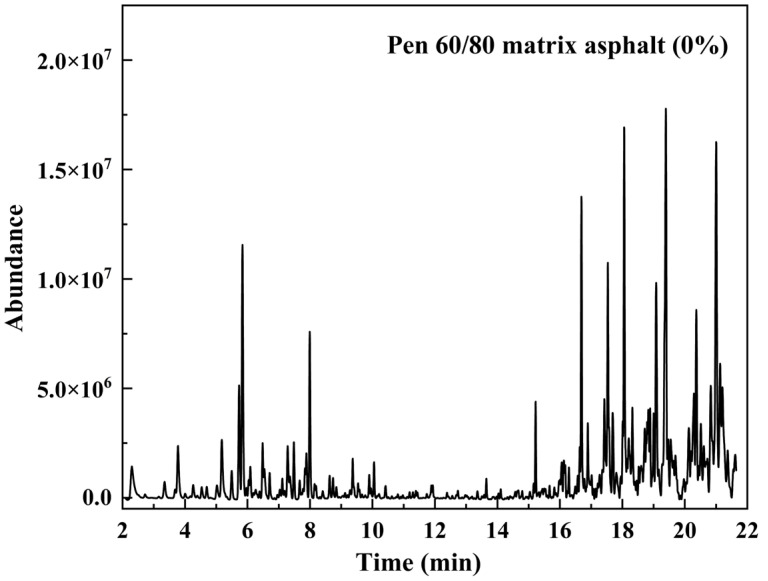
GC-MS pattern of matrix asphalt.

The thermal desorption chromatogram of the asphalt binder analyzed using TD-GC-MS is shown in Figure 5. The presence of each peak in the figure corresponds to a different substance. The name of each substance can be determined by comparison with the NIST 2014 MS library to perform a qualitative analysis of asphalt VOCs. The primary components of asphalt VOCs with a high matching degree and a large peak area are displayed in Table S1. By eliminating the effect of a small amount of air contained in the reaction device, the VOCs components can be roughly divided into three main categories, which represent aliphatic hydrocarbons (AH), benzene and its homologs (BH), hydrocarbon derivatives (HD) [38]. Here, hydrocarbon derivatives are designated as hydrocarbon compounds that include sulfur, nitrogen, and oxygen components. The principle of selecting components is substances with matching degrees for a more precise qualitative analysis. For the AH and BH kinds, which comprise the majority of VOCs, the substance is selected based on having a matching degree of more than 90. Furthermore, a substance with a matching degree of more than 70 is selected for the HD kinds that comprise a relatively small portion of the overall composition.

**Figure 6:**
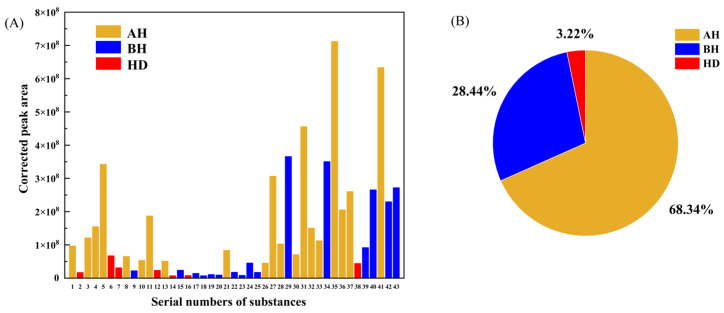
VOCs components from matrix asphalt: (**A**) Highly matched VOCs; (**B**) Proportion of asphalt VOC types.

As shown in Table S1, among the 43 listed substances, AH has the most kinds with 20 alkane or alkene types, while the types of BH and HD are 16 and 7, respectively. The histogram representation of the data presented in Table S1 can be seen in Figure 6A. The top three substances with corrected peak areas are the three substances numbered 31 tridecane, 35 tetradecane, and 41 pentadecane, all of which are chain hydrocarbons belonging to AH. Figure 6B illustrates the gas release proportion of three components in VOCs, with AH occupying the largest share of 68.34%, followed by BH at 28.44% and HD at 3.22%, respectively. Various aliphatic hydrocarbons exist in asphalt materials generated from the crude refinery, the chemical stability of which is significantly lower than that in benzene ring structures, so the release amount of AH will be higher than that of BH in asphalt VOCs [23].

#### 3.2.2. Adsorption Effect of Different ZC Dosage

In this study, various ZC dosages of 5 wt.% and 50 wt.% are prepared to analyze the rule of adsorption effect. The VOCs in the matrix asphalt addressed in the prior section has been designated as the control group for the blank, and the adsorption effect has been categorized into three different portions according to the organic category. The corrected area of a certain type was calculated by the sum of the corrected areas of different substances under the same type. The experiments are set to explore the difference between the large dosage and small dosage on VOCs adsorption effect of ZC and to try to analyze the possible reasons so as to lay the groundwork for the application of ZC on asphalt VOCs inhibition in road construction.

**Figure 7:**
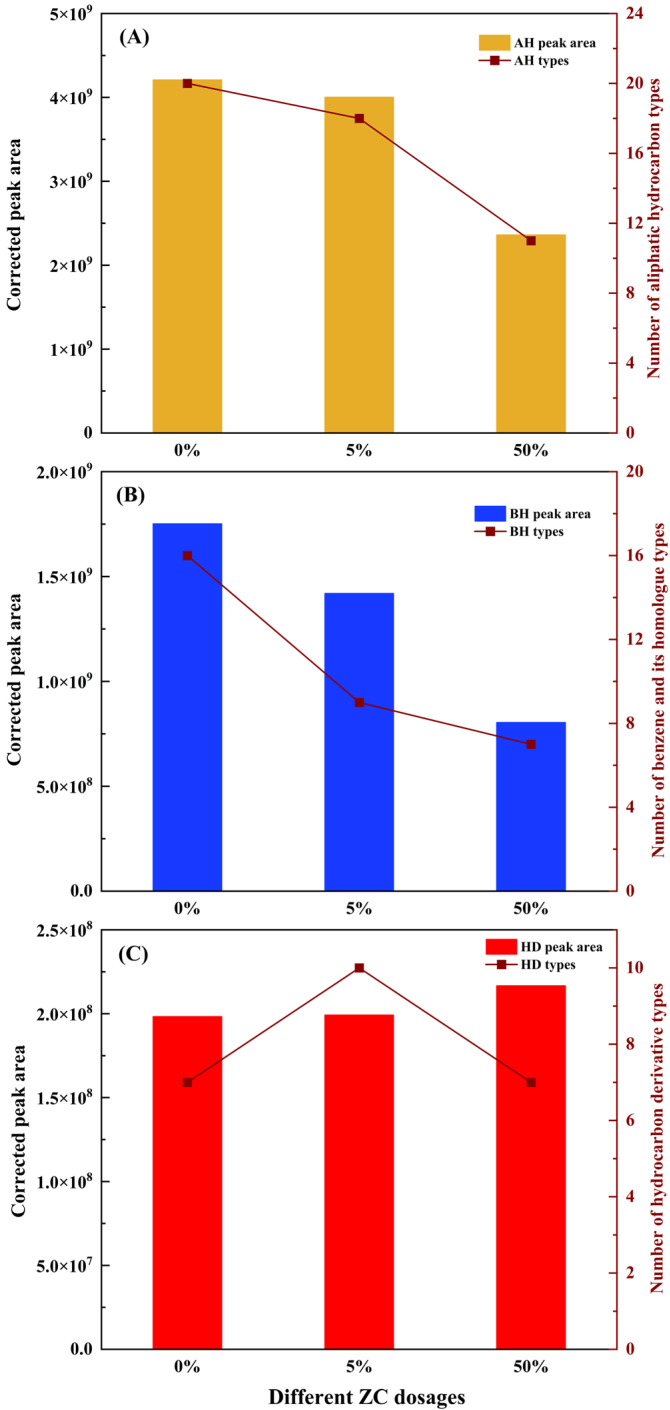
Adsorption effect of different asphalt VOCs types: (**A**) AH; (**B**) BH; (**C**) HD.

Figure 7 illustrates the influences of small doses of 5ZC and large doses of 50ZC on the adsorption effect of asphalt VOCs. As presented in Figure 7A, the peak area of AH exhibits a decreasing trend with the increase of incorporating amount. A conspicuous decrease can be observed at the dosage of 50%, which has decreased by 43.91% compared to the original situation. The number of AH types has a similar tendency to obtain the minimum types at the dosage of 50%, which is 45% less than the blank group. Figure 7B illustrates the adsorption effect on BH. Due to BH types containing the most carcinogenic PAHs, which are the source of emission of toxic volatile compounds, their inhibitory effect is of great significance. 5ZC and 50ZC both decrease in peak area and BH types compared to the blank group. 50ZC shows the largest reduction by 54.09% in the BH peak area, and the types decrease to 7, indicating 50ZC has an excellent adsorption effect on BH. Figure 7C shows that ZC has no obvious impact on the adsorption of HD. On the contrary, the peak area is increased by 9.29% at the dosage of 50%. Considering the raw materials and process of ZC preparation, some functional groups and chemical bonds present on the surface of ZC particles may stimulate HD generation in VOCs. Meanwhile, the selective adsorption of AH and BH by the internal pore structure of ZC impairs HD’s competitive adsorption capacity. Therefore, it increases the peak area of HD under the larger ZC incorporation amount. Increasing surface positive active sites of ZC might be profitable to improve the competitive adsorption capacity of HD.

To be brief, 50ZC performs satisfactorily in the adsorption effect of AH and BH, respectively, the primary component and the most toxic component in VOCs. 5ZC has a certain positive effect. VOCs components. Consequently, the effective way to reduce the emission of asphalt VOCs should be to incorporate ZC granules into the asphalt as fine aggregates rather than modified fillers.

#### 3.2.3. Adsorption Processes of Asphalt VOCs

Table 3 presents the adsorption effect of AH, BH, and the total adsorption effect of asphalt VOCs. The incorporation of ZC can manifestly suppress the emission of asphalt VOCs on the whole. At the dosage of 5%, the adsorption efficiency of particulate zeolite ceramsite on asphalt VOCs is significantly lower than that of powdered zeolite in a previous study [22]. The phenomenon that the adsorption capacity of powdered zeolite was greater than that of the granular form has also been reported [39]. On the one hand, it is reputed to have said that powdered zeolite would show a better adsorption effect since it has a higher surface area and, thus, more available adsorptive sites compared to granular zeolite [40,41,42]. On the other hand, the different adsorption capacities of ZC to molecules of different diameters in VOCs cannot be ignored [43]. Generally, the adsorption of VOCs onto ZC can be described in two steps: a rapid external mass transfer of VOCs molecules to the external surface of ZC through film diffusion and slow intraparticle diffusion of VOCs molecules into the interior of the ZC particles through pores [42]. When adsorbing small molecules, the molecules enter the pores through film diffusion and then start intraparticle diffusion. However, with the diffusion progressing, the molecules need to overcome the diffusion resistance so that they cannot reach the center of the particles. Eventually, the small molecules are stacked on the pore wall, decreasing the pore radius. Therefore, the macromolecular gases in VOCs cannot enter the pores and thus are released rather than absorbed. As for the adsorption of macromolecular gases in VOCs, macromolecules are also adsorbed on the external surface of ZC particles through film diffusion. But it is difficult or even impossible to enter the pores of adsorbents for internal diffusion due to their larger diameters. At the same time, the small molecules in VOCs will be hindered by the previously adsorbed macromolecules. Finally, only a shallow adsorption layer can be formed on the external surface of ZC particles. Therefore, the adsorption efficiency of 5ZC is not satisfactory on VOCs. The external surface area of ZC particles increases with the dosage rise; thus, 50ZC shows an ideal VOCs adsorption effect of 45.09%. However, further experiments are required to explore the optimal dosage for VOC adsorption. Moreover, it can be clearly observed from Table 3 in the results of the VOCs study that the adsorption effect of BH is about 10% higher than that of AH at both 5% and 50% dosages. The aromatic hydrocarbons in BH are macromolecules relative to the chain hydrocarbons in AH [16]. The highly selective adsorption of BH by ZC particles reduces the adsorption effect on AH, which also confirms the above processes from the side. Whereas to interpret the adsorption mechanisms more precisely, it is necessary to further characterize the ZC particles, including the judgment of pore structure type, specific surface area, pore cumulative volume, and pore size distribution.

### 3.3. Adsorption Mechanism of Asphalt VOCs by ZC

#### 3.3.1. Isotherms of ZC

**Figure 8:**
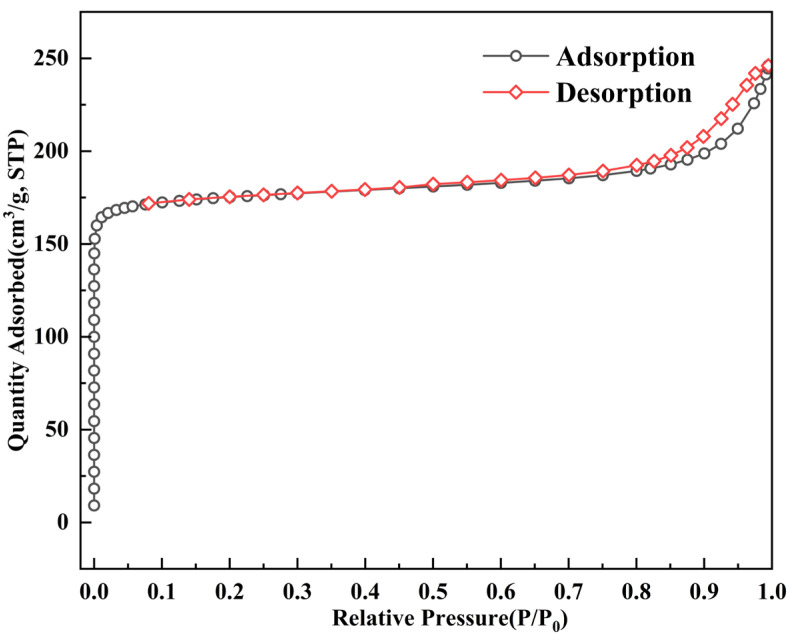
Nitrogen adsorption isotherm of ZC at 350 °C.

Figure 8 presents the nitrogen adsorption isotherm of ZC at a degasification temperature of 350 °C, which determines the type of pore structure. Nitrogen adsorption isotherm describes the connection between the maximum adsorbed quantity and balancing pressure at a given temperature. Adsorption isotherms can characterize the adsorption characteristics of different adsorbents. Currently, five types of adsorption isotherms are adopted prevalently in studying particle surface adsorption performance [44].

As shown in Figure 8, the curve characteristic of ZC presents a type I N2-adsorption isotherms, indicating the micropore domination within the sample. At low relative pressures (<0.1), due to the micropores being filled by the adsorbates, the nitrogen adsorption isotherm increased with a sharp gradient and was followed by the presence of a flat region in the isotherm. At a higher relative pressure (>0.8), the isotherm of the sample begins to increase sharply, indicating the presence of some textural mesopores [45]. In this relative pressure range, a weak hysteresis loop with type H3 can also be observed simultaneously. The obvious hysteresis loop is attributed to the filling of the larger mesopores and the occurrence of capillary condensation phenomena in mesopores [46,47]. The hysteresis loop with type H3 is a classic characteristic of aggregates (loose assemblages) of platelike particles forming slit-like pores [48], which may correspond to the component of ATP clay in ZC particles. All of these mentioned pores demonstrate that ZC prepared has formed the pore structure favorable for the internal diffusion of asphalt VOCs molecules.

#### 3.3.2. Pore Size Distribution of ZC

Currently, in terms of the pore size distribution (PSD), which plays a decisive role in the adsorption behaviors of porous materials, several methods for the calculation from adsorption data have been proposed [49]. However, the Barrett–Joyner–Halenda (BJH) method based on the Kelvin equation is suitable for mesoporous materials with a pore size larger than 5 nm [50]. In the micropore domain, the BJH method is not valid. Therefore, Horvath–Kawazoe (HK) method introduced a method for slit pores to calculate pore size and pore volume from isotherm data [51]. Saito–Foley (SF) method developed the cylindrical variant of the HK method, which has higher precision for estimating microporous PSD in zeolites [52].

**Figure 9:**
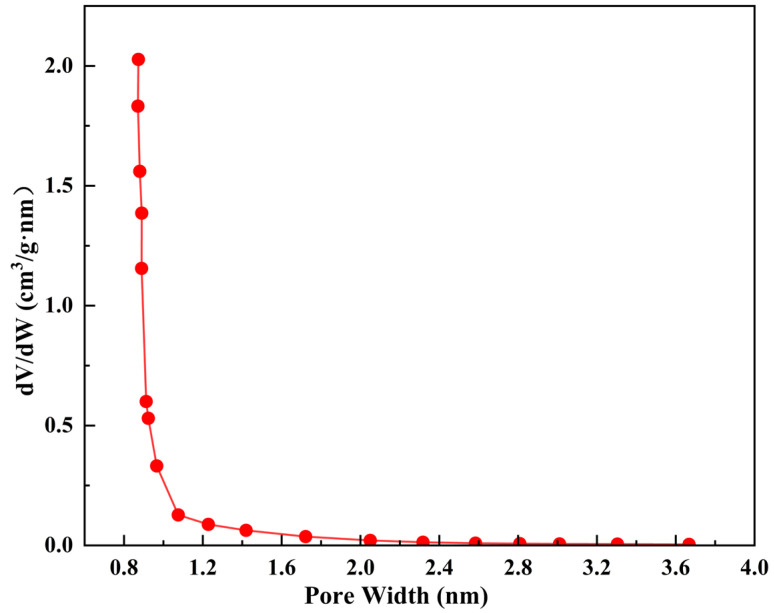
Pore size distribution of ZC.

Figure 9 shows the PSD of ZC based on the SF method. According to the SF calculation, PSD is primarily concentrated between 0.8 and 1 nm, which is consistent with the feature of the microporous material. The final calculation result indicates that the micropore size is 0.88 nm. Moreover, the other textural properties of ZC are listed in Table 4. ZC has a high total specific surface area of 709.34 m^2^/g at a pore volume of 0.38 cm^3^/g. Whereas it has a lower external specific surface area of 58.86 m^2^/g, which occupies 8.29% of the total specific surface area, indicating the surface adsorption of ZC particles is quite limited, and the intraparticle diffusion of asphalt VOCs molecules dominates the whole diffusion process. ZC has a micropore volume of 0.27 cm^3^/g, accounting for 71.05% of its total pore volume, and the remainder belongs to the interparticle mesopore volume mentioned above. The average pore size of 2.13 nm is obviously higher than that in micropore of 0.88 nm because it contains a small amount of mesopores stemming from ATP clay. Thus, the main pore structures in ZC can be classified as relatively smaller micropores.

Generally, the adsorption process by adsorbents is divided into physical adsorption and chemical adsorption. Chemical adsorption results from the chemical reaction between the surface functional groups of adsorbent and adsorbate molecules. Surface functional groups of porous materials are responsible for the chemical adsorption of VOCs [53]. The previous FTIR analysis shows that the surface functional groups of ZC do not nearly contain unsaturated atoms such as oxygen, sulfur, and nitrogen, indicating that physical adsorption dominates the adsorption process of ZC on asphalt VOCs. The physical adsorption of porous adsorbents mainly depends on specific surface area and pore structure. Meanwhile, based on the pore filling mechanism, the physical adsorption process of asphalt VOCs is close to the relationship between the diameter of the adsorbate molecule and the pore size of the adsorbent.

In the study of adsorbate molecules in zeolites, the molecular diameter is often characterized by the minimum kinetic diameter. Table 5 lists the kinetic diameter of some critical molecules of asphalt VOCs. The kinetic diameter of benzene is 0.59 nm, and PAHs contain several benzene rings. Thus, it can be speculated that the kinetic diameter is proportional to the number of benzene rings. The increase in the number of methyl groups also affects the kinetic diameter of the BH molecule. As for the AH category, the kinetic diameter of n-pentadecane, the longest chain hydrocarbon in asphalt VOCs, is 0.66 nm. Although the isomerism of normal-chain hydrocarbon molecules in AH will increase the kinetic diameter of molecules, the diameter of the aromatic hydrocarbon molecules in BH is still relatively large in general. The kinetic diameter of BH molecules is closer to the pore size distribution of 0.88 nm in ZC particles, indicating the pores have a firm adsorption capacity for BH molecules and are not accessible to desorption owing to the superposition of potential energy fields in adjacent wall pores [54]. When the pore size is larger than the kinetic diameter of molecules, a large amount of adsorption occurs in the pores due to capillary condensation. At the same time, the narrower micropore size of 0.88 nm can increase the diffusion resistance leading to low diffusion rates [55,56]. And the subsequent asphalt VOCs is hindered from diffusing into the intraparticle structure after capillary condensation. These two complex factors might be the reason for the high adsorption of BH. The above adsorption mechanisms of ZC are consistent with the previous discussion of the adsorption processes of asphalt VOCs.

## 4. Conclusions

In this study, ZC with a particle size of 2.36–1.70 mm was prepared under high-temperature calcination using commercial 13X zeolite powder as raw material and attapulgite clay as a binder. Then the prepared zeolite ceramsite was incorporated into the matrix asphalt as a modifier and fine aggregate at small and large dosages to explore their inhibitory effects on asphalt VOCs. Moreover, the structural characteristics of zeolite ceramsite were analyzed through a series of characterization tests to explain the inhibition mechanisms. The conclusions drawn are summarized as follows.

(1) The prepared zeolite ceramsite contains a trace of chemical components from the attapulgite binder through XRF analysis. Meanwhile, XRD and FTIR results did not detect the presence of obvious impurity crystal phases and atoms in the ZC, which possesses a high degree of crystallinity, indicating that the major crystal phase of the prepared product is 13X zeolite.
(2) The adsorption results of asphalt VOCs show that the volume reduction of total VOCs with high matching degrees exceeds 45% at a large dosage of 50%, indicating a significant VOCs inhibition effect. In comparison, with the addition at the dosage of 5%, the inhibitory effect of less than 10% is not ideal. Besides, the prepared zeolite ceramsite shows high selective adsorption of aromatic hydrocarbons in asphalt VOCs at both dosages, and their adsorption effect is about 10% higher than that of aliphatic hydrocarbons.
(3) The nitrogen adsorption isotherm indicates the micropore domination within the zeolite ceramsite and contains some textural mesopores stemming from the attapulgite binder. The micropore size of zeolite ceramsite is 0.88 nm, of which a micropore volume of 0.27 cm^3^/g accounts for 71.05% of its total pore volume. The main pore structures in ZC can be classified as relatively narrower micropores. The BET surface area is 709.34 m^2^/g, whereas the external specific surface area of 58.86 m^2^/g only occupies 8.29% of the total specific surface area, indicating that intraparticle adsorption dominates the whole adsorption process.
(4) The adsorption mechanism of asphalt VOCs by ZC is mainly physical adsorption. Under the narrow micropores within the material, the molecular kinetics diameter of most aromatic hydrocarbons is comparable to the pore size. The increase in the intraparticle diffusion resistance of aliphatic hydrocarbon molecules is found to be the important factor in obtaining high adsorption of aromatic hydrocarbons in asphalt VOCs.

This study attempts to reveal the feasibility of developing granular asphalt VOCs adsorbents through the effects of zeolite ceramsite on asphalt VOCs, and the results also indicate that granular adsorbents should be mixed into the asphalt as fine aggregates. However, the decline of asphalt mixture performance after the massive incorporation of adsorbents is an inevitable trend. Therefore, the focus of future work is to develop high-strength granular adsorbents that meet performance requirements for application in road construction.

## Figures and Tables

**Table 1:** Basic performances of the used asphalt binder.

Item	Results	Methods
Penetration (25 °C)/0.1 mm	63	ASTM D5
Softening Point/°C	51	ASTM D36
Ductility (15 °C)/cm	>100	ASTM D113
Density (15 °C)/g·cm^−3^	1.03	ASTM D8188
Solubility (Trichloroethylene)/%	99.7	ASTM D2042

**Table 2:** Main chemical composition of samples.

Oxides	Zeolite (%)	Attapulgite Clay (%)	Zeolite Ceramsite (%)
SiO_2_	41.02	52.06	42.38
Al_2_O_3_	30.01	12.36	25.06
Fe_2_O_3_	0.03	5.02	0.90
CaO	0.24	2.24	0.59
MgO	0.18	6.66	2.09
K_2_O	0.06	1.38	0.19
Na_2_O	15.84	0.56	12.48

**Table 3:** Adsorption efficiencies of asphalt VOCs.

Items	0ZC	5ZC	50ZC
AH types	20	17	11
AH volatilization	4209044861	4003208713	2361028006
AH adsorption effect	-	4.89%	43.91%
BH types	16	9	7
BH volatilization	1751970027	1519753368	804337964
BH adsorption effect	-	13.25%	54.09%
Total asphalt VOCs volatilization	6159307657	5722215181	3637487695
Total adsorption effect	-	8.72%	45.09%

**Table 4:** Textural properties of ZC sample.

Sample	S_BET_ (m^2^/g) ^a^	S_external_ (m^2^/g) ^b^	V_total_ (cm^3^/g) ^c^	V_micro_ (cm^3^/g) ^d^	Average Pore Size (nm) ^e^	Micropore Size (nm)
ZC	709.34	58.86	0.38	0.27	2.13	0.88

^a^ Total specific surface area S_BET_ was calculated by the BET method.; ^b^ External specific surface area S_external_ was calculated by the t-Plot method.; ^c^ Total pore volume V_total_ was calculated from nitrogen adsorption at P/P_0_ = 0.995.; ^d^ Micropore volume V_micro_ was calculated by the SF method.; ^e^ Average pore size of the sample was obtained from BET analysis.

**Table 5:** Reported values of kinetic diameter for some VOCs molecules.

Categories	Substances	Kinetic Diameter (nm)	Reference
AH	n-Hexane	0.45	[57]
	n-Octane	0.48	[57]
	Isooctane	0.62	[58]
	n-Pentadecane	0.66	[16]
BH	Benzene	0.59	[59]
	Toluene	0.59	[59]
	1,3,5-Trimethylbenzene	0.86	[59]
	2,6-Dimethylnaphthalene	0.72	[59]

## Data Availability

Not applicable.

## Supplementary Materials

The following supporting information can be downloaded at: https://www.mdpi.com/article/10.3390/ma15176100/s1, Microsoft Word v. 2016/2021 Table S1: Main substances of asphalt VOCs.
