# Supplementary material for: Adsorption Effect and Adsorption Mechanism of High Content Zeolite Ceramsite on Asphalt VOCs
Source: Materials (Basel). 2022 Sep 2;15(17):6100. doi: 10.3390/ma15176100 (PMC9457900; doi:10.3390/ma15176100)
Supplement: Supplementary file 1 [file materials-15-06100-s001.zip › materials-1859881-supplementary.pdf]

Table S1 Main substances of asphalt VOCs

Number	Time/min	Substance	Suitability	Peak area
1	3.776	C_5H_{12} (Pentane)	91	96557055
2	4.535	C_3H_6O (Acetone)	72	16961015
3	5.179	C_6H_{14} (2-Methylpentane)	91	121377299
4	5.733	C_6H_{12} (1-Hexene)	93	154499780
5	5.840	C_6H_{14} (Hexane)	94	342175671
6	6.486	C_4H_8O (Butanal)	95	67262589
7	6.710	C_4H_8O (2-Butanone)	86	31286879
8	7.486	C_7H_{16} (3-Methylhexane)	94	65190116
9	7.671	C_6H_6 (Benzene)	95	22157001
10	7.887	C_7H_{14} (1-Heptene)	97	53312432
11	7.993	C_7H_{16} (Heptane)	91	187110400
12	8.739	$C_5H_{10}O$ (Pentanal)	86	23500469
13	9.368	C_8H_{18} (2-Methylheptane)	97	50998377
14	9.585	$C_2H_6S_2$ (Dimethyl disulfide)	98	7286575
15	9.898	C_7H_8 (Toluene)	95	23769018
16	11.391	$C_3H_8S_2$ (Methyl ethyl disulfide)	94	7815323
17	11.886	C_8H_{10} (1,3-Dimethylbenzene)	97	14304660
18	12.387	C_8H_{10} (O-Xylene)	95	7325838
19	14.106	C_9H_{12} (1,3,5-Trimethylbenzene)	97	10787637
20	15.040	$C_{10}H_{14}$ (M-Cymene)	95	9376666
21	15.224	$C_{11}H_{24}$ (Undecane)	94	83552010
22	15.439	$C_{10}H_{14}$ (O-Cymene)	95	17577964
23	15.540	$C_{10}H_{14}$ (1-Ethyl-2,3-dimehtylbenzene)	96	8484848
24	16.129	$C_{10}H_{14}$ (1,2,4,5-Tetramethylbenzene)	91	45555904
25	16.492	$C_{10}H_{12}$ (2-Methyl-3-phenyl-1-propene)	91	17483172
26	16.636	$C_{12}H_{24}$ (1-Dodecene)	98	45323679
27	16.691	$C_{12}H_{26}$ (Dodecane)	96	306384349
28	16.895	$C_{13}H_{28}$ (2,6-Dimethylundecane)	96	102752304
29	17.536	$C_{10}H_8$ (Naphthalene)	95	365668351
30	18.013	$C_{14}H_{28}$ (1-Tetradecene)	98	70566200
31	18.060	$C_{13}H_{28}$ (Tridecane)	97	455454268
32	18.207	$C_{14}H_{28}$ (E-3-Tetradecene)	90	150607906
33	18.889	$C_{14}H_{30}$ (2-Methyltridecane)	90	112366936
34	19.084	$C_{11}H_{10}$ (1-Methylnaphthalene)	94	350629632
35	19.393	$C_{14}H_{30}$ (n-Tetradecane)	98	711859339
36	20.292	$C_{15}H_{32}$ (4-Methyltetradecane)	92	205359155
37	20.369	$C_{15}H_{32}$ (2,6,11-Trimethyldodecane)	90	260178985
38	20.446	$C_{14}H_{28}O$ (Tetradecanal)	83	44179919
39	20.609	$C_{12}H_{12}$ (2-Ethylnaphthalene)	93	91955162
40	20.831	$C_{12}H_{12}$ (2,6-Dimethylnaphthalene)	98	265363451
41	21.005	$C_{15}H_{32}$ (n-Pentadecane)	98	633418600
42	21.130	$C_{12}H_{12}$ (2,3-Dimethylnaphthalene)	98	229617912
43	21.204	$C_{12}H_{12}$ (2,7-Dimethylnaphthalene)	97	271912811
